# A Navigation Path Search and Optimization Method for Mobile Robots Based on the Rat Brain’s Cognitive Mechanism

**DOI:** 10.3390/biomimetics8050427

**Published:** 2023-09-14

**Authors:** Yishen Liao, Naigong Yu, Jinhan Yan

**Affiliations:** 1 Faculty of Information Technology, Beijing University of Technology, Beijing 100124, China; liaoyishen@emails.bjut.edu.cn (Y.L.); yjhcrossover@163.com (J.Y.); 2 Beijing Key Laboratory of Computational Intelligence and Intelligent System, Beijing 100124, China; 3 Engineering Research Center of Digital Community, Ministry of Education, Beijing 100124, China

**Keywords:** navigation path, optimization, mobile robots, boundary vector cells, place cells

## Abstract

Rats possess exceptional navigational abilities, allowing them to adaptively adjust their navigation paths based on the environmental structure. This remarkable ability is attributed to the interactions and regulatory mechanisms among various spatial cells within the rat’s brain. Based on these, this paper proposes a navigation path search and optimization method for mobile robots based on the rat brain’s cognitive mechanism. The aim is to enhance the navigation efficiency of mobile robots. The mechanism of this method is based on developing a navigation habit. Firstly, the robot explores the environment to search for the navigation goal. Then, with the assistance of boundary vector cells, the greedy strategy is used to guide the robot in generating a locally optimal path. Once the navigation path is generated, a dynamic self-organizing model based on the hippocampal CA1 place cells is constructed to further optimize the navigation path. To validate the effectiveness of the method, this paper designs several 2D simulation experiments and 3D robot simulation experiments, and compares the proposed method with various algorithms. The experimental results demonstrate that the proposed method not only surpasses other algorithms in terms of path planning efficiency but also yields the shortest navigation path. Moreover, the method exhibits good adaptability to dynamic navigation tasks.

## 1. Introduction

Navigation has always been one of the most important research topics in the field of mobile robotics. With the rapid development of sensor technology, computing power, and algorithms, the navigation capabilities of mobile robots have been significantly enhanced. However, the field of robot navigation still faces challenges such as low navigation efficiency, accuracy, and adaptability to the environment. Overcoming these challenges requires further research. Biological systems, through a long process of evolution, have developed efficient, flexible, and highly adaptive navigation mechanisms [1]. Through the interaction of mechanisms such as perception, memory, learning, and decision making, animals are able to excel at navigation tasks in various complex environments. Studying animal navigation abilities not only helps in deepening our understanding of biological mysteries but also provides valuable insights and inspiration for the field of robotics [2]. Therefore, many researchers have turned to the field of biomimicry, seeking inspiration to overcome the limitations of existing navigation methods.

As mammals, rats also possess remarkable navigation abilities. When rats are tasked with navigation in an unfamiliar environment, they rely on a series of perceptual, exploratory, and learning strategies to adapt to and address the navigation requirements of the new environment [3]. Firstly, rats explore the environment and search for the navigation goal. During this process, rats utilize their sensory systems and employ heuristic strategies to gather crucial information about the environment [4]. For example, they tend to follow walls, seek out bright lights, or avoid hazardous areas. These heuristic strategies help rats quickly acquire information about the environmental structure and safety, forming their cognitive understanding of the environment’s layout and available paths. Performing navigation tasks in an unfamiliar environment is a progressive adjustment and learning process, and rats cannot discover the optimal navigation strategy through a few explorations alone. However, rats possess learning capabilities and can gradually optimize their navigation paths through interactions with the environment [5]. When rats find that the current navigation path is not optimal, they may adjust their direction or adopt alternative strategies to obtain shorter and more direct paths. Additionally, changes in the environment can lead to the original navigation path being blocked or a shorter path appearing. In such cases, rats can establish a new navigation path adapted to the updated environment [6]. These adaptive and learning abilities enable rats to successfully cope with navigation challenges in unfamiliar environments and gradually achieve efficient navigation.

To uncover the mechanisms underlying rat navigation abilities, physiologists have conducted in-depth studies on the neural circuits, activity patterns of neurons, and interactions between brain regions in the rat brain. They have found that the entorhinal-hippocampal structure is a crucial brain region for rat positioning and navigation [7,8]. Specifically, the entorhinal-hippocampal structure receives perceptual information from other brain regions, such as visual, olfactory, and spatially relevant information [9]. The hippocampus is primarily involved in the encoding and integration of spatial memory [10]. Within the entorhinal-hippocampal structure, there are various types of neurons (spatial cells) that exhibit specific firing patterns related to spatial locations, including place cells(PCs) [11], grid cells(GCs) [12], boundary vector cells(BVCs) [13], and head direction cells [14]. Self-motion information is believed to be input to the grid cell structure in the entorhinal cortex [15] and projected through neural networks to the place cells in the hippocampal CA3 region, enabling path integration [16]. During rat exploration, obstacles and boundaries in the environment are primarily encoded by boundary vector cells in the entorhinal cortex. The position information encoded by hippocampal CA3 place cells is then projected to hippocampal CA1 place cells, facilitating the storage and memory of spatial information [17]. There are also complex networks of interactions among hippocampal CA1 place cells. These interactions can be achieved through mechanisms such as neuronal firing and synaptic plasticity, promoting self-organizing activity among place cells [18]. The self-organizing characteristics of hippocampal CA1 place cells are believed to play a crucial role in optimizing navigation paths. Through the self-organizing process, hippocampal CA1 place cells can adjust their own activity patterns and location specificity based on the experiences gained during rat exploration.

In summary, establishing a bionic navigation method based on the firing mechanisms of spatial cells is crucial for achieving efficient navigation. Based on this, this paper proposes a mobile robot navigation path search and optimization method inspired by the cognitive mechanisms of the rat brain. The mechanism of this method is based on developing the navigation habit, and it has made progress in the following aspects:

1. With the assistance of the boundary vector cells’ firing model, a navigation path search method based on the greedy strategy is proposed. This method can guide the robot to generate locally optimal paths based on the firing activity of boundary vector cells.
2. A dynamic self-organizing model based on hippocampal CA1 place cells is established to further optimize the navigation path and improve navigation performance.
3. Two-dimensional simulation experiments and three-dimensional robot simulation experiments demonstrate the advantages of the proposed method in terms of navigation path length and path planning efficiency (the number of explorations required to find the optimal navigation path). Furthermore, the method exhibits strong adaptability to environmental changes and navigation tasks. When navigation tasks or the environment changes, it can discover a new path faster than other algorithms.

The following sections are organized as follows: Section 2 describes related research in the field of navigation. Section 3 presents the detailed principles of the proposed method. Section 4 describes the experiments and results. Finally, Section 5 and Section 6 provide the discussion and conclusions of this paper.

## 2. Related Works

The path planning and navigation of mobile robots have been widely studied, and classic path planning methods mainly include the A * algorithm [19], the Rolling-Window RRT algorithm [20], etc. However, these types of algorithms have a strong dependence on heuristic functions and parameter selection, and do not have good adaptability to dynamic navigation tasks. In recent years, research on spatial navigation methods based on biomimetic cognitive mechanisms has gained increasing attention from researchers, primarily in two research directions. The first direction is biomimetic environment map construction and navigation. This research aims to construct environmental cognitive maps based on the operating principles of the rat brain and then perform path planning and navigation based on cognitive maps. Yu et al. simulated the computational models of four types of spatial cells in the entorhinal-hippocampal structure using a unified computational mechanism, enabling the construction of environmental cognitive maps and navigation based on these models [21]. Liu et al. proposed a self-organizing learning framework based on episodic memory for robot experiential learning, cognitive map construction, and navigation [22]. However, although map-based path planning and navigation methods are widely used in practical applications, they often tend to be more engineering-oriented and lack biomimetic fidelity to the real navigation abilities of biological systems.

Another direction focuses on the development of navigation habits based on biomimetic cognitive mechanisms. This research aims to develop navigation models that guide robots to explore their spatial environment and gradually acquire navigation habits. As the number of times the robot explores the environment increases, it can spontaneously learn and plan paths for specific navigation tasks. These methods directly target specific navigation tasks, eliminating the need to explore the entire environment for the purpose of constructing a cognitive map. Furthermore, these navigation methods align more closely with the characteristics of animal navigation. Oudeyer et al. [23] proposed an intelligent adaptive curiosity-driven learning theory that enables robots to explore without prior knowledge and gradually achieve environmental cognition. Ruan et al. [24] constructed an environmental cognition model based on the theory of curiosity-driven learning and implemented path planning for mobile robots. Research on the development of navigation habits based on biomimetic cognitive mechanisms can be traced back to 2009 when Kulvicius et al. [25] used a simple feedforward neural network to establish connections between hippocampal place cells and action neurons. They employed the Q-learning algorithm to adjust the neural network and achieve goal-directed navigation for agents. Subsequently, Frémaux et al. [26] utilized spiking neural networks as the connection structure between place cells and action neurons, incorporating spike-timing-dependent plasticity (STDP) learning rules to adjust the network’s connection weights, thus improving the speed of the navigation model in discovering the navigation goal. To enable intelligent agents to navigate efficiently to dynamic reward locations, Zannone et al. [27,28] introduced the sequential neuromodulation mechanism of acetylcholine and dopamine into the STDP learning rule, enhancing the adaptability of the model. However, research on the neural mechanisms that support dynamic adjustment of navigation paths with environmental changes is still limited in the aforementioned methods. Therefore, DeepMind developed a deep learning network based on LSTM (Long Short-Term Memory) networks and applied it to pathfinding tasks in virtual maze environments, achieving vector-based navigation encoded by grid cells [29]. This model can discover shortcuts in the environment and exhibits good generalization, flexibility, and adaptability. However, this model requires significant training time and a large-scale neural network, making it less suitable for mobile robots.

## 3. Materials and Methods

### 3.1. Overall Operation Mechanism of the Method

**Figure 1:**
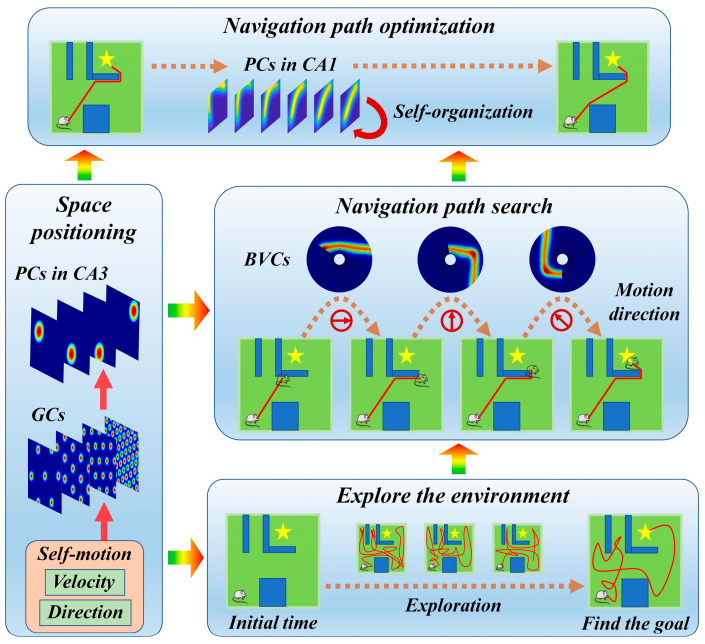
The overall operational mechanism of the method.

This subsection provides a detailed introduction to the navigation path search and optimization method based on the rat brain’s cognitive mechanism. It consists of four main components: spatial localization, environment exploration, navigation path search, and navigation path optimization. Firstly, the robot is allowed to explore the environment freely in order to find the navigation goal. Once the robot discovers the navigation goal, the greedy strategy is employed to search for the navigation path. In this stage, boundary vector cells play a crucial role in recognizing the environment boundaries and obstacles. Finally, the dynamic self-organizing computational model of hippocampal CA1 place cells is used to optimize the navigation path. This model can adaptively adjust the navigation path based on the environmental structure. Spatial localization serves as the foundation for the entire process. Grid cells and hippocampal CA3 place cells integrate self-motion cues to obtain the robot’s position information within the environment [30]. The overall operational mechanism of the method is illustrated in Figure 1.

### 3.2. Navigation Path Search Method Based on Boundary Vector Cells and Greedy Strategy

This paper employs a greedy strategy to guide the robot in searching for a navigation path. Due to the presence of numerous obstacles in the environment, the robot needs to possess obstacle-avoidance capabilities. The recognition function of the boundary vector cell can assist the robot in avoiding collisions with obstacles and finding shorter paths toward the goal. The boundary vector cell exhibits a Gaussian-tuned response to the presence of obstacles, reaching its peak at preferred distances and orientations [31]. The firing rate of the boundary vector cell, denoted as $F_{BVC}$, is mathematically expressed as follows:

(1) $$F_{BVC}=g(d,\theta )\delta \theta$$

In Equation (1), δθ represents the angular range of the boundary vector cell’s receptive field. g(d,θ) describes the relationship between the firing rate and the angle and distance, and its mathematical expression is as follows:

(2) $$g(d,\theta )\propto \frac{\exp\left[-{\left(d-d_{bvc}\right)}^{2}/2\sigma_{\mathrm{rad}}^{2}\left(d_{bvc}\right)\right]}{\sqrt{2\pi \sigma_{\mathrm{rad}}^{2}}}\times \frac{\exp\left[-{\left(\theta -\phi \right)}^{2}/2\sigma_{\mathrm{ang}}^{2}\right]}{\sqrt{2\pi \sigma_{\mathrm{ang}}^{2}}}$$

In Equation (2), $d_{bvc}$ and ϕ represent the preferred distance and preferred direction of the boundary vector cell, respectively. d represents the distance between the robot and the obstacle, and θ represents the actual bearing angle of the obstacle relative to the robot. $\sigma_{\mathrm{rad}}$ and $\sigma_{\mathrm{ang}}$ represent the distance tuning factor and angle tuning factor, respectively, and they are both constants. When using a greedy strategy, the robot selects the next movement direction based on its current position and the activity of the boundary vector cell. Let $r_{t}$ denote the position of the robot in the environment at time t. The mathematical expression for the robot’s position at the next time $r_{t+1}$ is as follows:

(3) $$r_{t+1}=r_{t}+[v\cdot cos(\alpha_{t}),v\cdot sin(\alpha_{t})]$$

In Equation (3), v represents the robot’s movement speed. $\alpha_{t}$ denotes the angle of the next movement direction, determined by the greedy strategy and the firing activity of the boundary vector cell. Firstly, discretize all obstacles in the environment and define the set of discretized obstacle positions as OBS. The mathematical expression is as follows:

(4) $$OBS=\{{obs}^{1},{obs}^{2},\ldots ,{obs}^{k}\}$$

**Figure 2:**
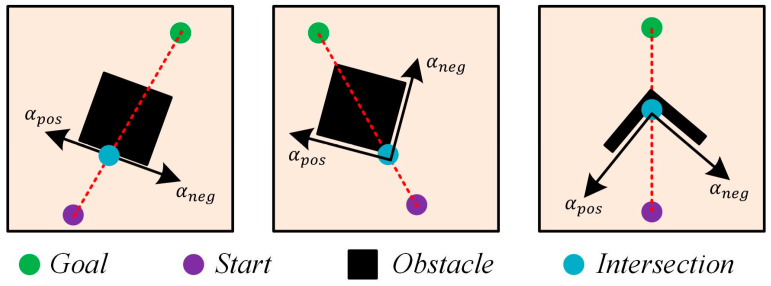
The diagrams illustrating the angles $\alpha_{pos}$ and $\alpha_{neg}$.

When the robot detects an obstacle in its movement direction, the boundary vector cells generate firing activity. In this case, the greedy strategy will provide two possible movement directions for the robot, denoted as $\alpha_{pos}$ and $\alpha_{neg}$, as shown in Figure 2.

The function f(α) is defined to represent the absolute difference between α and $\alpha_{obj}$. Its mathematical expression is as follows:

(5) $$f(\alpha )=\left|Delt(\alpha ,\alpha_{obj})\right|$$

In Equation (5), “Delt” represents the calculation of the difference between two angles, and $\alpha_{obj}$ represents the azimuth angle of the goal relative to the robot. Based on this, the mathematical expressions for $\alpha_{pos}$ and $\alpha_{neg}$ are as follows:

(6) $$\alpha_{pos}=\underset{Delt(\alpha ,\alpha_{obj})\in \left(0,\pi \right)}{\arg min}f(\alpha )$$

(7) $$\alpha_{neg}=\underset{\;Delt(\alpha ,\alpha_{obj})\in \left(-\pi ,0\right)}{\arg min}f(\alpha )$$

Next, the calculation steps for determining the robot’s next movement direction $\alpha_{t}$ are provided. Firstly, the angle selection function $h\left(\alpha ,\beta \right)$ is defined, which represents the smaller value between the angle differences of $\alpha_{pos}$ and $\alpha_{neg}$ with respect to $\alpha_{obj}$. The mathematical expression for $h\left(\alpha ,\beta \right)$ is as follows:

(8) $$h\left(\alpha ,\beta \right)=\left\{\begin{array}{c} \alpha_{pos}\;\;\;f(\alpha_{pos})\leq f(\alpha_{neg})\\ \alpha_{neg}\;\;\;f(\alpha_{pos})>f(\alpha_{neg}) \end{array}\right.$$

The set of boundary positions is defined as BORDER, and the set of all previously visited locations by the robot is denoted as PATH. Let $r_{t+1}(\alpha )$ represent the next moment position of the robot when it moves in the direction α. Next, we classify and discuss the different cases for the values of $\alpha_{t}$. Since both the starting point and the target are within the environment, the robot moving along the $\alpha_{obj}$ direction will never collide with obstacles. Therefore, the statement $r_{t+1}(\alpha_{obj})\notin BORDER$ always holds. When $r_{t+1}(\alpha_{obj})\notin OBS$, it means that the robot can move along the $\alpha_{obj}$ direction without colliding with obstacles. According to the greedy strategy, in this case, the robot will directly choose $\alpha_{obj}$ as its movement direction. On the other hand, when $r_{t+1}(\alpha_{obj})\in OBS$, it implies that the robot will collide with an obstacle if it moves along the direction $\alpha_{obj}$. In this situation, the mathematical expression for calculating $\alpha_{t}$ is as follows:

(9) $$\alpha_{t}=\left\{\begin{array}{c} h\left(\alpha ,\beta \right)\;\;\;\;r_{t}\notin PATH\;\;AND\;\;\;r_{t+1}(h\left(\alpha ,\beta \right))\notin PATH\\ \hat{h}\left(\alpha ,\beta \right)\;\;\;\;\;\;r_{t}\in PATH\;\;OR\;\;\;r_{t+1}(h\left(\alpha ,\beta \right))\in PATH\;\; \end{array}\right.$$

In Equation (9), the function $\hat{h}\left(\alpha ,\beta \right)$ represents selecting a value from $\alpha_{pos}$ and $\alpha_{neg}$ that is not equal to $h\left(\alpha ,\beta \right)$. Once the value of $\alpha_{t}$ is determined, if $r_{t+1}(\alpha_{t})\in BORDER$, it means that the robot will collide with the boundary if it moves in the selected direction $\alpha_{t}$. In this case, the robot will return along the same path. It means that $\alpha_{t}={\pi +\alpha }_{t-1}$ at this time. Through these steps, the robot can progressively move towards the destination guided by the greedy strategy, completing the path planning task.

### 3.3. Dynamic Self-Organizing Model of Hippocampal CA1 Place Cells

However, the navigation paths obtained from the greedy strategy tend to be more curved and further optimization is required. After the hippocampal CA1 place cells store the navigation paths, they can adaptively adjust the paths based on the environmental structure. This adjustment is achieved through the self-organizing mechanism of the place cells. Let the *i*-th place cell be denoted as $e^{i}=\{\beta^{i},p^{i}\}$, where $p^{i}=(p_{x}^{i},p_{y}^{i})$ represents the center coordinates of the firing field. $\beta^{i}$ represents the orientation angle of the agent at $p^{i}$, initially set as the angle between the line connecting the firing field centers of adjacent place cells $e^{i}$ and $e^{i+1}$ and the positive *x*-axis. The update in the position of the *i*-th place cell’s firing field center at time t is denoted as $∆p^{i}(t)=(∆p_{x}^{i}(t),∆p_{y}^{i}(t))$, and the correction to the orientation angle is denoted as ${∆\beta }^{i}(t)$. The relevant mathematical expressions are as follows:

(10) $$∆p_{x}^{i}\left(t\right)=p_{x}^{i}\left(t\right)+{\tau d}^{i}\cos\left(\beta^{i}\left(t\right)+\varphi_{i}\right)$$

(11) $$∆p_{y}^{i}\left(t\right)=p_{y}^{i}\left(t\right)+{\tau d}^{i}\sin\left(\beta^{i}\left(t\right)+\varphi_{i}\right)$$

(12) $${∆\beta }^{i}(t)=Delt(\beta^{i+1}\left(t\right),\beta^{i}\left(t\right))$$

In Equations (10)–(12), τ represents the relaxation factor, $d^{i}$ represents the distance between the firing field centers of place cells $e^{i}$ and $e^{i+1}$ at the initial time, and $\varphi_{i}$ represents the difference between $\beta_{i}$ and $\beta_{i+1}$ at the initial time. After obtaining the correction values, the firing field centers and orientation angles can be adjusted. The mathematical expression for the correction of the firing field center coordinates is as follows:

(13) $$p^{i}\left(t+1\right)=p^{i}\left(t\right)+\delta \left(p^{i+1}\left(t\right)-∆p^{i}\left(t\right)\right)$$

(14) $$p^{i+1}\left(t+1\right)=p^{i+1}\left(t\right)-\delta \left(p^{i+1}\left(t\right)-∆p^{i}\left(t\right)\right)$$

The mathematical expression for head orientation angle correction is as follows:

(15) $$\beta^{i}\left(t+1\right)=\beta^{i}\left(t\right)+\delta {∆\beta }^{i}(t)$$

(16) $$\beta^{i+1}\left(t+1\right)=\beta^{i+1}\left(t\right)-\delta {∆\beta }^{i}(t)$$

In Equations (13)–(16), δ represents the correction gain. However, in practical physical environments, there are often many obstacles, so the optimization process of the path also needs to consider the influence of obstacles. Therefore, in this study, the path optimization process is divided based on the firing mechanism of boundary vector cells. Let $l_{min}^{i}(t)$ represent the shortest distance between the firing field center of the *i*-th place cell and the obstacles at time t. Its mathematical expression is as follows:

(17) $$l_{min}^{i}(t)=min(\left\|p^{i}\left(t\right)-{obs}^{k}\right\|)$$

During the process of optimizing the navigation path, when the firing field center of a particular place cell gradually approaches an obstacle and the distance between them becomes sufficiently small, the firing field center of the place cell will be fixed. Let $P_{fix}\left(t\right)$ represent the set of place cells with fixed firing field centers at time t. When t=0, $P_{fix}\left(t\right)=\{p^{0}\left(t\right),p^{N_{hpc}}\left(t\right)\}$. The mathematical expression for the update of $P_{fix}\left(t\right)$ with increasing iterations is as follows:

(18) $$P_{fix}\left(t+1\right)=sort(P_{fix}\left(t\right)\cup \left\{p^{i\in i_{fix}}\left(t+1\right)\right\})$$

(19) $$i_{fix}=\left\{l_{min}^{i}(t+1)\leq l_{th}|l_{min}^{i}(t+1)-l_{min}^{i}(t)<0\right\}$$

**Figure 3:**
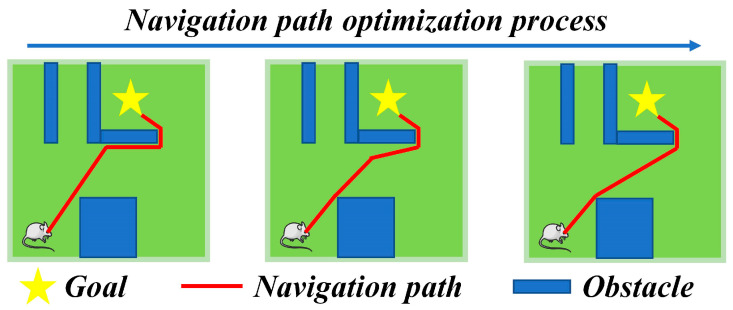
Operation mechanism of navigation path segment optimization.

In Equations (18) and (19), “sort” represents sorting the elements in the set in ascending order, and $l_{th}$ represents the threshold for the shortest distance criterion. Through the aforementioned steps, the navigation path can be segmented and corrected, ensuring efficient target-oriented navigation in environments with obstacles. The operational mechanism of segmented correction for the navigation path is illustrated in Figure 3.

### 3.4. Proof of Convergence of the Self-Organizing Computational Model

To demonstrate the theoretical correctness of the self-organizing computational model, we demonstrate the convergence of the model. The proof process is as follows. Firstly, the total energy function at time t is defined as $E\left(t\right)$, and its mathematical expression is as follows:

(20) $$E\left(t\right)=E_{x}\left(t\right)+E_{y}\left(t\right)$$

In Equation (20), $E_{x}\left(t\right)$ and $E_{y}\left(t\right)$ represent the components of energy $E\left(t\right)$ along the *x*-axis and *y*-axis, respectively. It is evident that the convergence or divergence of $E_{x}\left(t\right)$ is consistent with $E_{y}\left(t\right)$. Therefore, it is sufficient to prove the convergence of $E_{x}\left(t\right)$ as t→∞ to establish the convergence of $E\left(t\right)$. The mathematical expression for $E_{x}\left(t\right)$ is as follows:

(21) $$E_{x}\left(t\right)=E_{x}^{i+1\to i}\left(t\right)+E_{x}^{i-1\to i}\left(t\right)$$

In Equation (21), $E_{x}^{i-1\to i}\left(t\right)$ and $E_{x}^{i+1\to i}\left(t\right)$ represent the sum of energy functions along the *x*-axis from the (*i* − 1)-th and (*i* + 1)-th place cells to the *i*-th place cell, respectively. Their mathematical expressions are as follows:

(22) $$E_{x}^{i-1\to i}\left(t\right)=\sum_{i=2}^{N_{hpc}}{\left(\left\|p_{x}^{i}\left(t\right)-∆p_{x}^{i-1}\left(t\right)\right\|\right)}^{2}$$

(23) $$E_{x}^{i+1\to i}\left(t\right)=\sum_{i=1}^{N_{hpc}-1}{\left(\left\|p_{x}^{i+1}\left(t\right)-∆p_{x}^{i}\left(t\right)\right\|\right)}^{2}$$

As the starting and ending points of navigation are fixed, the energy values of the first and last place cells do not change. Let $C_{i-1}^{i}=p_{x}^{i-1}\left(t\right)+d^{i-1}\cos\left(\beta^{i-1}\left(t\right)+\varphi_{i-1}\right)$ and $C_{i+1}^{i}=p_{x}^{i+1}\left(t\right)-d^{i}\cos\left(\beta^{i}\left(t\right)+\varphi_{i}\right)$. In this case, the mathematical expressions for $E_{x}^{i-1\to i}\left(t\right)$ and $E_{x}^{i+1\to i}\left(t\right)$ are transformed as follows:

(24) $$E_{x}^{i-1\to i}\left(t\right)=\sum_{i=2}^{N_{hpc}}{\left(\left\|C_{i-1}^{i}-p_{x}^{i}\left(t\right)\right\|\right)}^{2}$$

(25) $$E_{x}^{i+1\to i}\left(t\right)=\sum_{i=1}^{N_{hpc}-1}{\left(\left\|C_{i+1}^{i}-p_{x}^{i}\left(t\right)\right\|\right)}^{2}$$

Next, the change in $E_{x}^{i-1\to i}\left(t\right)$ at time t, denoted as $∆E_{x}^{i-1\to i}\left(t\right)$, is calculated Each iteration process is essentially the displacement of the node towards the point of minimum energy, which is the expectation of all points in the set. The mathematical expression for $∆E_{x}^{i-1\to i}\left(t\right)$ is as follows:

(26) $$∆E_{x}^{i-1\to i}\left(t\right)={\left(\left\|C_{i-1}^{i}-Exp(C_{i-1}^{i})\right\|\right)}^{2}-{\left(\left\|C_{i-1}^{i}-p_{x}^{i}\left(t\right)\right\|\right)}^{2}$$

In Equation (26), “Exp” represents the expectation function. Since $C_{i-1}^{i}$ is a constant value, we have $Exp(C_{i-1}^{i})=C_{i-1}^{i}$. As the energy function is defined based on the relative positions of the points rather than their absolute coordinates, the absolute position of the firing field center of the place cell does not affect the change in the energy function. Assuming $p_{x}^{i}\left(t\right)=0$, the mathematical expression for $∆E_{x}^{i-1\to i}\left(t\right)$ can be transformed as follows:

(27) $$∆E_{x}^{i-1\to i}\left(t\right)=-{\left(C_{i-1}^{i}\right)}^{2}$$

Similarly, the mathematical expression for $∆E_{x}^{i+1\to i}\left(t\right)$ can be transformed as follows:

(28) $$∆E_{x}^{i+1\to i}\left(t\right)=-{\left(\left\|C_{i+1}^{i}-p_{x}^{i}\left(t\right)\right\|\right)}^{2}=-{\left(C_{i+1}^{i}\right)}^{2}$$

From Equations (27) and (28), it can be concluded that $∆E_{x}^{i-1\to i}\left(t\right)\leq 0$ and $∆E_{x}^{i+1\to i}\left(t\right)\leq 0$. According to Equation (21), it follows that the change in the energy function along the *x*-axis, $∆E_{x}\left(t\right)\leq 0$. By following the same steps, it can be shown that the change in the energy function along the *y*-axis, $∆E_{y}\left(t\right)\leq 0$. Therefore, as t→∞, the energy function $E\left(t\right)$ converges.

### 3.5. Dynamic Navigation Tasks

**Figure 4:**
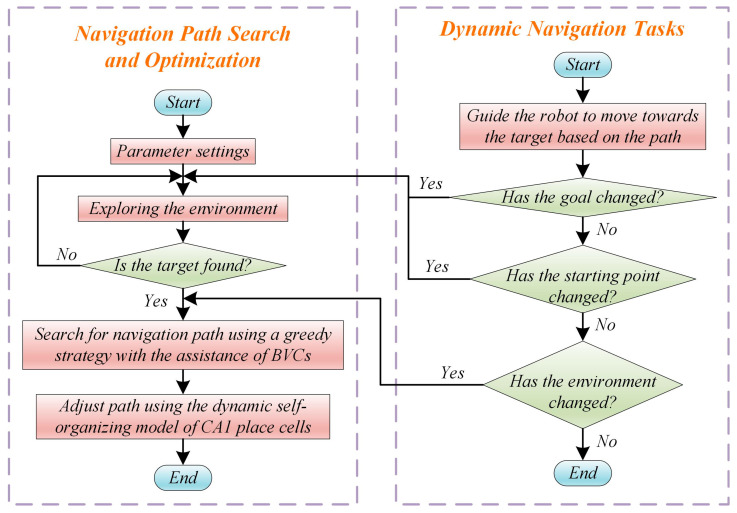
Operation process of the navigation method.

When a robot is in a static environment, it only needs to explore the environment and search for the navigation path. However, spatial environments or navigation tasks are often constantly changing, requiring the robot to have strong adaptability. Changes in the environment can occur in two main situations: (1) The original navigation path is blocked due to the addition of obstacles in the environment. (2) As the robot progresses along its pre-planned navigation path, obstacles that were previously detectable by the robot may no longer be detected. This situation represents a reduction in obstacles in the environment. Additionally, the navigation task itself may change, mainly involving changes in the starting and ending points. When the navigation endpoint changes, the robot needs to explore the environment again to search for a new navigation path. When the navigation starting point or the environment changes, the robot only needs to search for a new navigation path. The overall process of the navigation method is illustrated in Figure 4, and the specifics of the proposed navigation method can be found in Algorithm 1.
**Algorithm 1** The proposed navigation method1: **Initialize** population and parameters 2: **while** not find the navigation goal **do**
3:   **while** The maximum path has not been reached **do**
4:     **Update** $r_{t+1}=r_{t}+[v\cdot cos(\alpha_{t}),v\cdot sin(\alpha_{t})]$, the value of $\alpha_{t}\;is$ randomly select 5:   **end while**
6: **end while**
7: **while** not find the navigation goal **do**
8:   **Calculate** the value of $\alpha_{obj}$
9:   **Calculate** the firing rate of boundary vector cells using Equations (1) and (2) 10:   **if** (the firing rate of boundary vector cells did not reach the threshold) **then**
11: **Update** $\alpha_{t}=\alpha_{obj}$
12:   **else**
13: **Calculate** the value of $\alpha_{pos}$, $\alpha_{neg}$, and $h\left(\alpha ,\beta \right)$ using Equations (6)–(8) 14: **Update** the value of $\alpha_{t}$ using Equation (9) 15: **end if**
16: **Update** $r_{t+1}=r_{t}+[v\cdot cos(\alpha_{t}),v\cdot sin(\alpha_{t})]$
17: **end while**
18: **while** not reach the number of iterations for optimization **do**
19: **Calculate**
$l_{min}^{i}(t)=min(\left\|p^{i}\left(t\right)-{obs}^{k}\right\|)$
20: **if (**$l_{min}^{i}(t)\leq l_{th}$ and $l_{min}^{i}(t+1)-l_{min}^{i}(t)<0$**) then**
21: **Update** the elements in sets $P_{fix}$ and $i_{fix}$ using Equations (18) and (19) 22:   **end if**
23: **Calculate** the value of $∆p_{x}^{i}\left(t\right)$, $∆p_{y}^{i}\left(t\right)$, and ${\;∆\beta }^{i}(t)$ using Equations (10)–(12) 24: **Update** the values of all path coordinates $p^{i}\left(t+1\right)$ using Equations (13)–(16) 25: **end while**
26: **Return** the set of all path coordinates $p^{i}\left(t+1\right)$ for the optimal navigation path 

## 4. Experiments and Results

In this section, we experimentally validate the navigation path search and optimization method. The experiments include 2D navigation experiments, dynamic navigation experiments, and 3D robot simulation experiments. The 2D simulation experiments are conducted in the MATLAB environment, while the robot experiments are conducted in the Webots simulation environment. The computer configuration used for the experiments is as follows: Windows 11 operating system, Intel Core i7-11800H CPU, 16GB DDR4 memory, and NVIDIA RTX 3060 graphics card. The parameter settings for the navigation method are as follows: the robot’s movement speed v is set to 0.1 m/s, the correction gain δ is set to 0.5, and the threshold for the shortest distance criterion $l_{th}$ is set to 0.2 m. The experimental parameters for the boundary vector cells are selected based on reference [32], and the experimental parameters for the entorhinal-hippocampal spatial localization model are selected based on reference [30].

### 4.1. 2D Navigation Experiments

In this section, we validate the performance of the proposed method through 2D simulation experiments. Four different spatial regions are constructed for navigation experiments, with each region having an area of 10 m × 10 m. Obstacles are set within the spatial region and the starting point and goal for navigation are defined.

#### 4.1.1. Path Search Experiment

**Figure 5:**
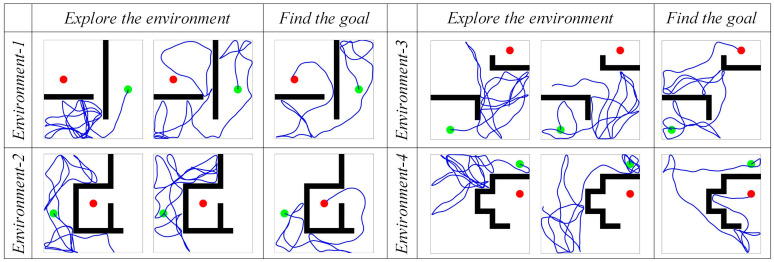
The motion trajectories of the agent in the spatial regions. In the figures, the green circle represents the navigation starting point, the red circle represents the navigation endpoint, the black wall represents the obstacle, and the blue line represents the robot’s motion trajectory.

First, the agent is allowed to explore the environment and search for the navigation goal. The maximum path length for a single exploration process is set to 80 m. The motion trajectories of the agent in the spatial regions are shown in Figure 5. The sequence from left to right represents the increasing number of exploration attempts. The green and red circles represent the starting and ending points of navigation, respectively. The black rectangles represent obstacles, and the blue lines represent the movement paths of the agent in the spatial regions. It can be observed that as the agent moves continuously, its trajectory covers the entire spatial environment. When the robot reaches the navigation goal, it records its own position as the position of the navigation goal.

**Figure 6:**
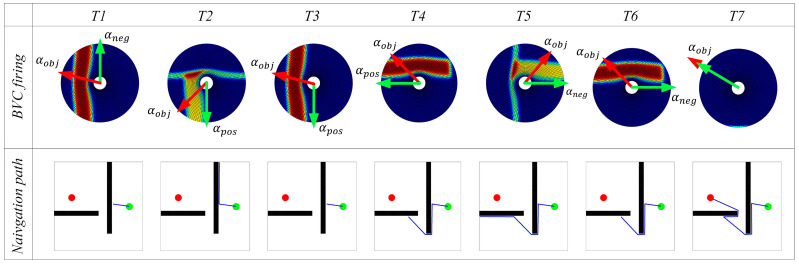
Experimental results of path searching process of the agent in environment 1. In the figures, the green circle represents the navigation starting point, the red circle represents the navigation endpoint, the black wall represents the obstacle, the blue line represents the robot’s navigation path, and the columns represent successive time points during the simulation.

Once the robot discovers the navigation goal, it uses the greedy strategy assisted by the boundary vector cell to search for the navigation path. Figure 6 shows the variation of the navigation path and the firing activity of the boundary vector cell during the path search process in environment 1. From Figure 6, it can be observed that with the assistance of the boundary vector cell, the agent is able to use the greedy strategy to search for the navigation path.

**Figure 7:**
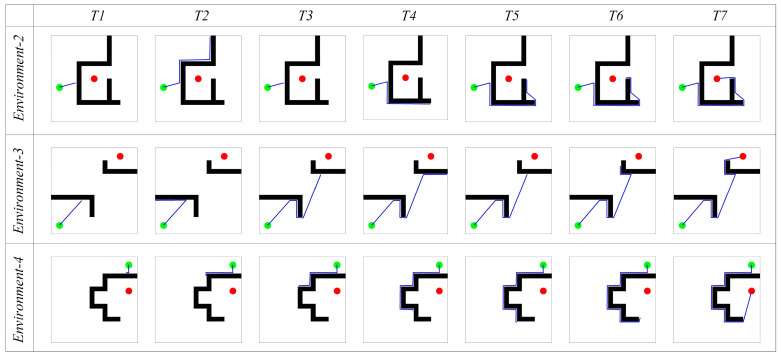
Experimental results of path searching process of the agent in environment 2, 3, and 4. In the figures, the green circle represents the navigation starting point, the red circle represents the navigation endpoint, the black wall represents the obstacle, the blue line represents the robot’s navigation path, and the columns represent successive time points during the simulation.

The red arrow in Figure 6 represents the $\alpha_{obj}$ direction, and the green arrow represents the next movement direction given by the path search algorithm. Time T1 and T4 represent the moments when the agent first encounters an obstacle. The direction with the smallest angle difference with $\alpha_{obj}$ from $\alpha_{pos}$ and $\alpha_{neg}$ is selected as the next movement direction. In the subsequent movement process, if the agent detects the boundary of the environment, it returns along the same path, as shown in time T2 and T5 in Figure 6. Then, in time T3 and T6, the agent returns to the position where it first encountered the obstacle, selects the other direction from $\alpha_{pos}$ and $\alpha_{neg}$ as the next movement direction, and continues to search for the navigation path. If the boundary vector cell does not perceive any obstacles in the movement direction, the agent directly selects $\alpha_{obj}$ as the next movement direction, as shown in time T7 in Figure 6. The path search is then performed in environments 2, 3, and 4, with the results shown in Figure 7. The experimental results demonstrate that the proposed method can accurately search for the navigation path.

#### 4.1.2. Path Optimization Experiment

**Figure 8:**
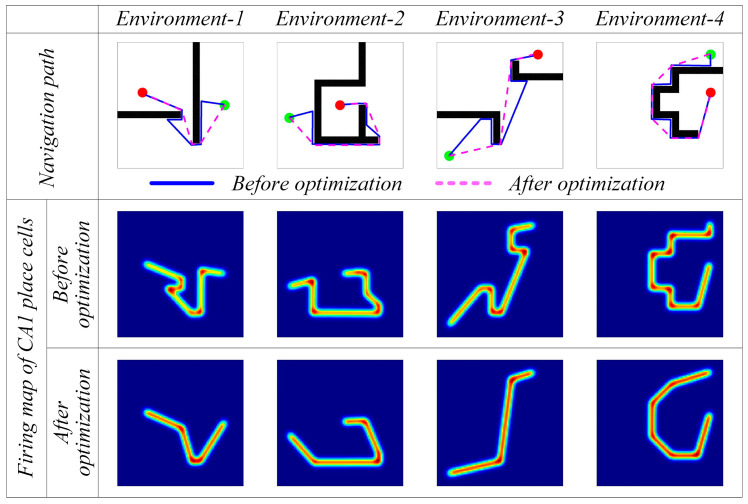
Experimental results before and after navigation path optimization. In the figures, the green circle represents the navigation starting point, the red circle represents the navigation endpoint, the black wall represents the obstacle, the blue line represents the robot’s navigation path before optimization, and the pink dashed line represents the robot’s navigation path after optimization.

However, from Figure 6 and Figure 7, it can be observed that the navigation paths obtained by the greedy strategy are somewhat curved and may not be optimal. Therefore, further optimization is needed using the hippocampal CA1 place cells’ self-organizing model. Figure 8 shows the results before and after navigation path optimization. The first row represents the comparison of the paths before and after optimization, where the blue lines represent the original paths and the pink dashed lines represent the optimized navigation paths. The second row represents the firing rate map of the CA1 place cells before path optimization, where brighter colors indicate a stronger firing activity of the CA1 place cells in that area. The third row represents the firing rate map of the CA1 place cells after path optimization.

**Figure 9:**
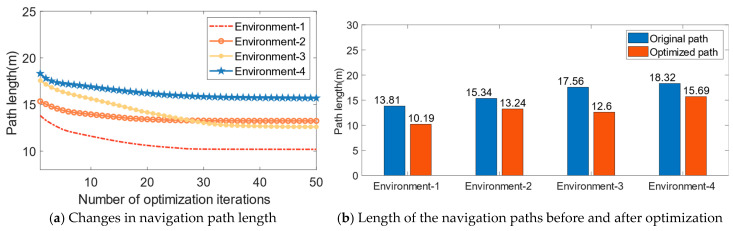
Path length results for navigation path optimization.

Figure 9a shows the change in path length during the optimization process, and Figure 9b presents the length statistics of the navigation paths before and after optimization. From Figure 8 and Figure 9, it can be seen that with an increasing number of iterations, the optimized paths are significantly shorter compared to the original paths, validating the effectiveness of the CA1 place cell self-organizing model for navigation path optimization.

#### 4.1.3. Comparative Experiment

In order to further highlight the advantages of the proposed method, it is compared with two classic path planning algorithms, the A * algorithm [19] and the Rolling-Window RRT algorithm [20]. Due to the different operating mechanisms between these types of algorithms and the navigation habit development algorithms, only the path length of the algorithm is compared. To avoid randomness, each algorithm was run twenty times in each environment and the average value was calculated as the experimental result. The path length results are shown in Table 1. As it can be seen from the table, compared to the A * algorithm and the Rolling-Window RRT algorithm, the proposed method generates the shortest navigation path, verifying the effectiveness of the method.

**Figure 10:**
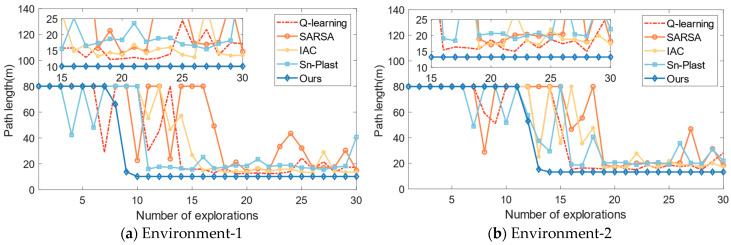

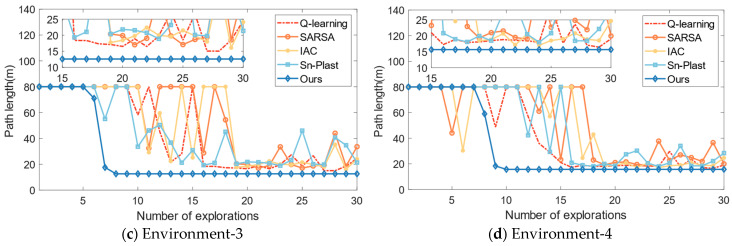
The variation of path length for each algorithm with increasing exploration iterations.

Then, in order to further highlight the advantages, it is compared with several navigation habit development algorithms, such as the Q-learning algorithm [25], the SARSA algorithm [33], the Sn-Plast algorithm [28], and the Intelligent Curiosity Algorithm (IAC) [23]. These algorithms are all reinforcement learning algorithms that can guide the agent to explore the spatial environment and develop navigation habits. The number of explorations in a single navigation habit formation process is set to 30, and the maximum path length for a single exploration process is set to 80 m. The variation of path length with the number of explorations is shown in Figure 10.

From Figure 10, it can be observed that all algorithms are able to guide the agent in developing navigation habits in the corresponding space. The SARSA algorithm has the worst navigation performance, with a slower convergence speed and longer navigation path lengths compared to the other algorithms. The Q-learning algorithm outperforms the SARSA, Sn-Plast, and IAC algorithms in terms of navigation path length, but it discovers the navigation goal slower than the Sn-Plast algorithm. However, although Sn-Plast can discover the navigation goal faster, it fails to converge in a timely manner. This leads to a situation where the goal is discovered in the current exploration process but cannot be reached in the next exploration. On the other hand, the agent using the proposed method can quickly converge once the target is discovered and navigate steadily to the goal in the subsequent exploration tasks. Furthermore, the navigation paths formed by the proposed method are also the shortest among all algorithms.

In order to further investigate the advantages of the proposed model in terms of convergence speed and navigation path, comparative experiments were designed in terms of the average length of navigation paths, the probability of convergence after discovering the navigation goal, and the average number of explorations required to complete the formation of navigation habits. To quantify the convergence performance of the model, the following criteria are used: when the agent discovers the goal in the *n*-th exploration and is able to discover the target in the n + 1 to n + 3 explorations, the model is considered to have converged (indicating the formation of navigation habits). The probability of convergence after discovering the target region, denoted as $P_{con}$, is mathematically expressed as follows:

(29) $${\;P}_{con}={Sum}_{con}/{Sum}_{find}$$

In Equation (29), ${Sum}_{find}$ represents the number of times the navigation goal is discovered, and ${Sum}_{con}$ represents the number of times the navigation goal is discovered and the agent is able to navigate to the goal in the subsequent three exploration tasks. The average length of navigation paths, denoted as $L_{avg}$, represents the average value of the exploration path lengths for all the times the navigation goal is discovered, and it is mathematically expressed as follows:

(30) $$L_{avg}=\sum_{j=1}^{{Sum}_{find}\;}L_{j}/{Sum}_{find}$$

In Equation (30), $L_{j}$ represents the exploration path length for the *j*-th time the navigation goal is discovered. The average number of explorations required to complete the formation of navigation habits represents the average number of explorations required for the algorithm to converge, which reflects the speed of guiding the agent to discover the target region and the convergence speed. Without loss of generality, each experiment is conducted 20 times, and the experimental results of navigation for each algorithm are shown in Table 2.

The experimental results in Table 2 once again demonstrate that the proposed method outperforms other algorithms in terms of the probability of convergence after discovering the navigation goal, convergence speed, and navigation path length, confirming the superiority of the algorithm. Subsequently, an analysis and discussion of the experimental results can be provided. For the various compared reinforcement learning algorithms, the speed of developing navigation habits is influenced by two factors: firstly, the speed of discovering the navigation goal, which refers to the efficiency of the agent in finding the navigation goal in the environment; and secondly, the convergence speed after discovering the navigation goal, which pertains to how quickly the agent can form a stable memory of the optimal navigation path once the goal is found. However, for the proposed method, the speed of developing navigation habits is solely determined by the speed of discovering the goal. The proposed method is capable of converging immediately once the agent discovers the goal, allowing for the rapid formation of stable navigation habits even during initial encounters with the goal.

#### 4.1.4. Dynamic Navigation Experiments

**Figure 11:**
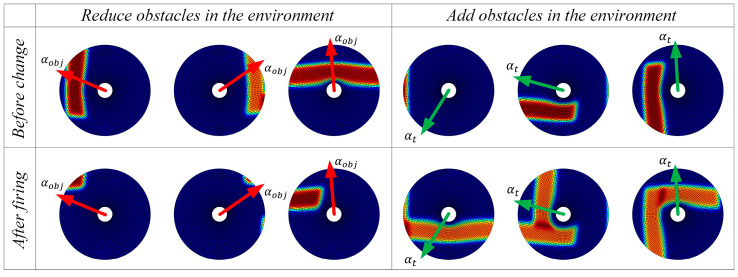
Firing effects of the boundary vector cell sensing the changes in obstacles.

**Figure 12:**
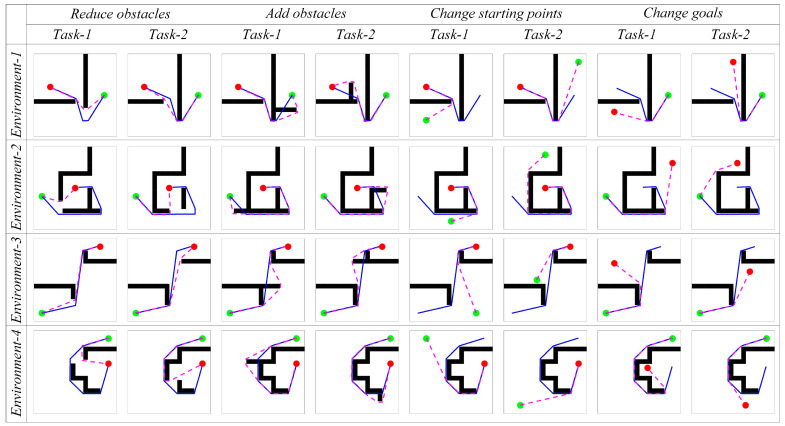
The adjustment effect of navigation paths in dynamic navigation tasks. In the figures, the green circle represents the navigation starting point, the red circle represents the navigation endpoint, the black wall represents the obstacle, the blue line represents the robot’s original navigation path, and the pink dashed line represents the robot’s new navigation path.

**Figure 13:**
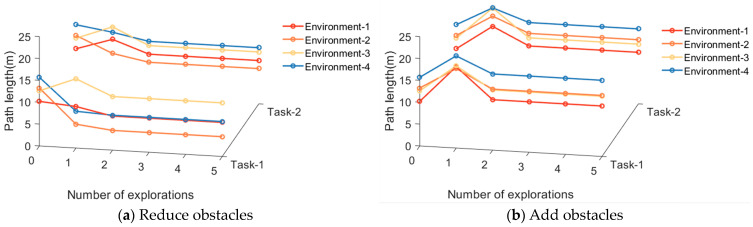

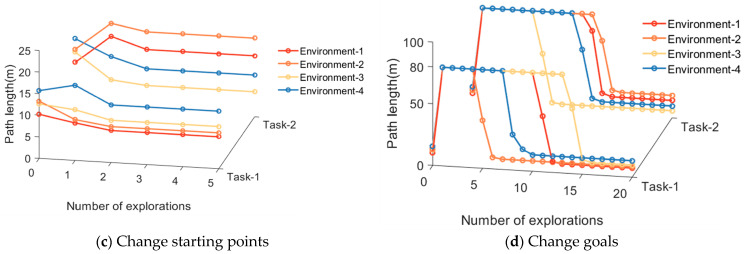
The variation of path length in dynamic navigation experiments.

The adaptability of the model to dynamic changes in navigation tasks is verified by adding or removing obstacles on the original navigation path and changing the starting and ending points of the navigation task. The behavior of the algorithm in response to environmental changes and changes in navigation tasks is observed. The changes in obstacles in the environment are primarily perceived through the boundary vector cell. When the agent moves along the navigation path, if obstacles are detected in the direction $\alpha_{t}$, the boundary vector cell indicates that obstacles have been added to the environment and the agent needs to detour. If no obstacles are detected in the direction $\alpha_{obj}$, the boundary vector cell indicates that obstacles have been removed from the environment. The firing effects of the boundary vector cell sensing the changes in obstacles are shown in Figure 11. The adjustment effect of navigation paths in dynamic navigation tasks is shown in Figure 12, and the change in path length with exploration iterations is shown in Figure 13.

From the experimental results, it can be observed that the proposed method exhibits rapid adaptation to dynamic navigation tasks and is capable of adjusting to environmental and task changes. Through the perception of boundary vector cells, the agent can detect changes in obstacles and accordingly avoid new obstacles or find shorter navigation paths. This ability is crucial for dealing with uncertainty and dynamic changes in real-world navigation tasks, and provides strong support for the agent to demonstrate more powerful navigation capabilities in practical applications.

Then, the average number of exploration iterations required to relearn navigation habits in dynamic navigation tasks is compared among different algorithms, including the proposed method and classical reinforcement learning algorithms. The experimental parameters remain consistent with the previous experiments. The results are shown in Table 3. From the table, it can be observed that the proposed method has the fastest convergence speed. Moreover, when the navigation goal remains unchanged, the agent only requires one exploration iteration to generate a new navigation path.

Subsequently, the experimental results are analyzed and discussed. For the compared reinforcement learning algorithms, it is necessary to explore the environment and develop new navigation habits whenever there are changes in the environment or navigation tasks. However, for the proposed method, it only needs to explore the environment again and find the new navigation goal when the goal changes. In other cases, the agent can directly search for a new navigation path guided by the greedy strategy, greatly improving the speed of adapting to new environments or navigation tasks.

### 4.2. Robot Experiments on the 3D Simulation Platform

**Figure 14:**
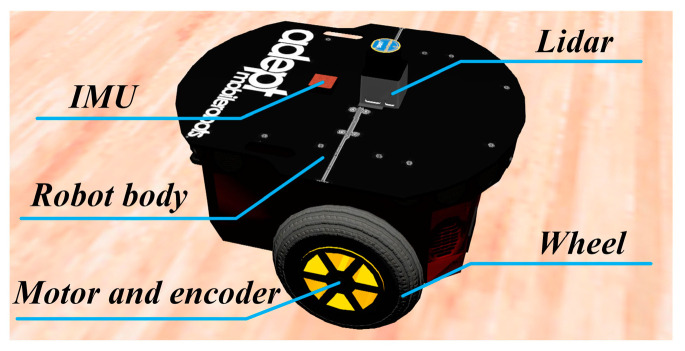
Physical structure diagram of the mobile robot.

To further validate the effectiveness of the method, navigation experiments were conducted using a robot platform in the Webots simulation environment. The robot platform in the Webots simulation environment is illustrated in Figure 14. The Pioneer-3DX robot was used as the experimental platform. This robot has omnidirectional mobility and uses a wheeled chassis and electric drive system for motion control. Additionally, the robot is equipped with various sensors, including a lidar for detecting obstacles and boundaries of the environment, an IMU for obtaining the robot’s orientation information, and encoders for measuring the robot’s linear velocity during motion.

**Figure 15:**
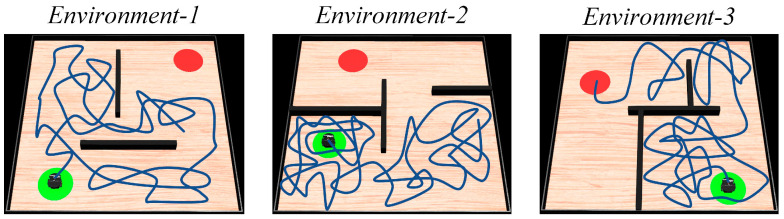
Mobile robot’s motion trajectory during the exploration process. In the figures, the green circle represents the navigation starting point, the red circle represents the navigation endpoint, the black wall represents obstacles, and the blue line represents the robot’s motion trajectory.

**Figure 16:**
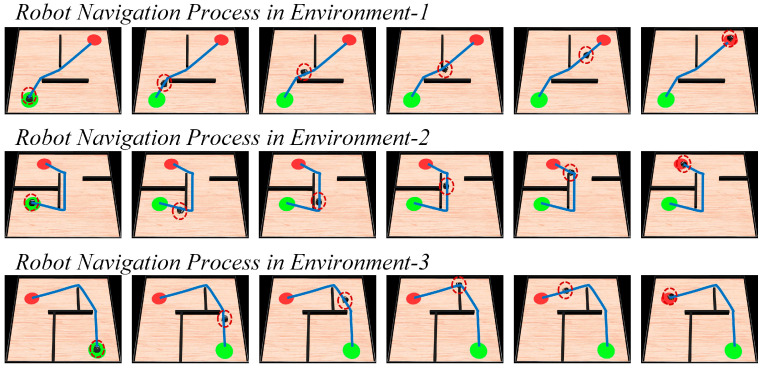
The process of the robot navigating towards the goal. In the figures, the green circle represents the navigation starting point, the red circle represents the navigation endpoint, the black wall represents the obstacle, the blue line represents the robot’s motion trajectory, and the red dashed circle represents the robot’s current position.

**Figure 17:**
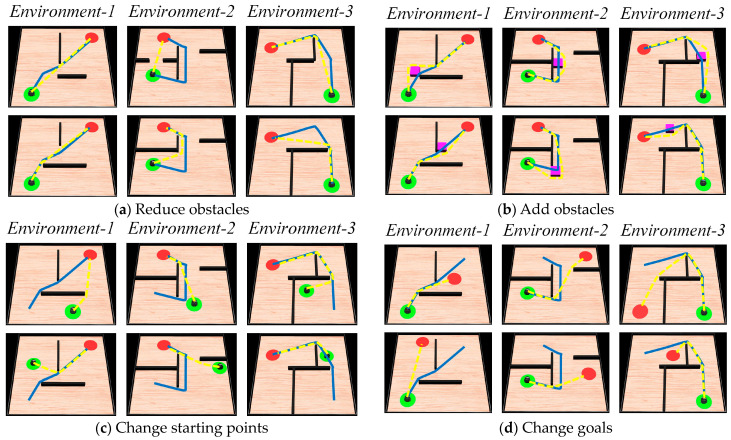
Motion process of the robot completing dynamic navigation tasks. In the figures, the green circle represents the navigation starting point, the red circle represents the navigation endpoint, the black wall represents the obstacle, the blue line represents the robot’s original navigation path, the yellow dashed line represents the new navigation path, and the pink cube represents new obstacles.

The experiments are conducted in three different simulated environments, each with a size of 10 m × 10 m. The environments are randomly set up with starting points, target points, and obstacles such as walls and blocks. The parameters of the method are kept consistent with the previous description. The next position coordinates for the robot are calculated using the proposed method, and then the robot is controlled to move to the specified positions, and guided to explore the environment. The motion trajectory of the robot during the exploration of the environment is shown in Figure 15. Once the navigation path is generated, the robot follows the trajectory to move towards the navigation goal, as illustrated in Figure 16. Subsequently, following the design of the 2D experiment, the robot is tasked with performing dynamic navigation tasks in the environment. The experimental results are shown in Figure 17.

In Figure 15, the blue line represents the robot’s motion trajectory, the red region represents the navigation goal, and the green color represents the starting point. In Figure 16, the sequence from left to right represents the robot’s movement from the starting point to the navigation goal, and the red dashed circle represents the robot’s current position. In Figure 17, the blue line represents the original navigation path, while the yellow dashed line represents the newly generated navigation path. From Figure 15 and Figure 16, it can be observed that as the robot explores the environment, it is able to discover the target and generate the navigation path to guide itself towards the goal accurately. Figure 17 shows that when there are changes in the environment, the robot can use its lidar to perceive the changes and adjust the navigation path accordingly. When the navigation starting point changes, the robot can directly use the proposed method to re-plan the navigation path. Similarly, when the navigation goal changes, the robot can re-explore the environment and re-plan the navigation path. The robot platform experiment further validates the navigation performance of the proposed method and emphasizes its practical applicability.

## 5. Discussion

In this paper, a navigation path search and optimization method based on the rat brain’s cognitive mechanism was established, which provides a possible explanation for the navigation mechanism in the rat brain. The running steps of the method are as follows. Firstly, the robot is allowed to explore the environment freely in order to find the navigation goal. Once the robot discovers the navigation goal, the greedy strategy is employed to search for the navigation path. In this stage, boundary vector cells play a crucial role in recognizing the environment boundaries and obstacles. Finally, the dynamic self-organizing computational model of hippocampal CA1 place cells is used to optimize the navigation path.

From the previous section, it can be seen that the proposed model can demonstrate excellent navigation performance. Firstly, in 2D navigation experiments, the proposed method was compared with two classic path planning algorithms, the A * algorithm and the Rolling-Window RRT algorithm. The experimental results show that the proposed method generates the shortest navigation path. The main reason for this result is that these types of algorithms have a strong dependence on heuristic functions and parameter selection, and their path-planning length is also affected by the initial motion angle. However, the proposed method does not require setting too many parameters, and can generate the optimal navigation path regardless of the initial motion angle. Then, the proposed method was compared with several navigation habit development algorithms. The experimental results demonstrate that the proposed method outperforms other algorithms in terms of the probability of convergence after discovering the navigation goal, convergence speed, and navigation path length. The main reason for this result is that the proposed method is capable of converging immediately once the agent discovers the goal, allowing for the rapid formation of stable navigation habits even during initial encounters with the goal. On the contrary, other navigation habit development algorithms require a learning process when discovering the navigation target and cannot converge immediately.

However, spatial environments or navigation tasks are often constantly changing, requiring the robot to have strong adaptability. Therefore, dynamic navigation experiments were also designed to test the adaptability of the method. The experimental results demonstrate that the proposed method has the fastest convergence speed. The other algorithms need to explore the environment and develop new navigation habits whenever there are changes in navigation tasks. However, the proposed method only needs to explore the environment again and find the new navigation goal when the goal changes. In other cases, the agent can directly search for a new navigation path.

To further test the method, navigation experiments were conducted using a robot platform in the 3D simulation environment. The experimental results show that the proposed method can drive real robots to perform excellent navigation. However, the robot experiments conducted in this paper only involve driving the robot to move in 2D space, and it is currently not suitable for higher-dimensional space navigation. Firstly, for navigation, obtaining the robot’s position in the environment is the most important task. In the proposed method, the entorhinal-hippocampal CA3 neural computing model is used for positioning. It contains various firing models of spatial cells, such as hippocampal CA3 place cells and grid cells. Secondly, in the path searching and optimization stages, the proposed method also uses the firing models of boundary vector cells and hippocampal CA1 place cells. All the above spatial cells can only exhibit firing characteristics in 2D space in current physiological research, and these spatial cells only have firing rate mathematical models in 2D space. In fact, recent physiological research has found that grid cells in the rat brain can exhibit firing characteristics in 3D space [34]. Therefore, constructing the mathematical models of 3D spatial cells and applying them to navigation in high-dimensional space will be the objective of future work.

In addition, although the proposed method can find short paths, it must be admitted that sometimes the shortest path is not the least costly path. For example, a path that is not the shortest can become the least costly path in a tailwind situation. Therefore, the optimal navigation path needs to comprehensively consider various influencing factors, rather than just the shortest distance [35]. In future work, this issue will be taken into account to achieve more efficient navigation methods.

## 6. Conclusions

This paper proposes a navigation path search and optimization method for mobile robots based on the rat brain’s cognitive mechanism, and the mechanism of this method is based on developing a navigation habit. In order to validate the method, this paper conducts a series of 2D and 3D robot simulation experiments. By comparing with various other algorithms, the experimental results demonstrate the significant advantages of the proposed method in terms of path planning efficiency and shortest navigation path. The main conclusions are as follows:

- This study draws inspiration from the navigation abilities of rats. It successfully applies the simulation of the interactions and regulatory mechanisms among various spatial cells in the rat brain to search and optimize the navigation path of mobile robots. This demonstrates the potential of the rat brain’s cognitive mechanism in solving complex navigation problems.
- During the exploration phase, the use of a greedy strategy and the assistance of boundary vector cells guide the robot to search for locally optimal navigation paths. Subsequently, a dynamic self-organizing model based on hippocampal CA1 place cells is constructed to further optimize the navigation paths and improve navigation efficiency.
- The proposed method not only exhibits advantages in terms of navigation paths and path planning efficiency but also demonstrates strong adaptability to environmental and navigation task changes, allowing for the rapid generation of paths that adapt to new navigation tasks.

However, the proposed method also has some limitations: 1. In physical environments, robot motion often involves cumulative errors, which are not considered in the proposed method and may affect the navigation performance due to imprecise localization. 2. If the spatial environment is complex, the greedy search-based approach may not obtain the globally optimal path for the current navigation task.

Therefore, future research directions include the following: 1. Applying the proposed method to real robot systems and incorporating various kinds of external information as input to improve robot localization during the robot–environment interaction process, thereby enhancing the robustness of the model. 2. Exploring inspirations from cognitive mechanisms in different animals and applying them to robot navigation, which can provide new insights and methods for innovation in navigation algorithms.

In summary, the research findings of this paper lay the foundation for a robot navigation method based on the imitation of the rat brain’s cognitive mechanism and have significant implications for the field of mobile robotics.

## Figures and Tables

**Table 1:** Comparison results of path length with A* algorithm and Rolling-Window RRT algorithm.

Algorithm	Average Navigation Path Length (m)			
	Environment-1	Environment-2	Environment-3	Environment-4
A * algorithm	10.91	13.89	13.07	16.11
Rolling-Window RRT	11.23	14.36	12.92	16.34
**Ours**	**10.19**	**13.24**	**12.60**	**15.69**

**Table 2:** Statistical results of navigation experiments of various algorithms.

Environment	Algorithm	$L_{avg}$(m)	$P_{con}$	Average Number of Explorations Required to Develop Navigation Habits
1	Q-learning	13.12	83.8%	13.2
	SARSA	14.78	70.5%	16.7
	IAC	13.71	81.1%	15.9
	Sn-Plast	15.06	71.5%	12.3
	**Ours**	**10.19**	**100%**	**8.4**
2	Q-learning	17.96	85.4%	15.8
	SARSA	19.10	58.9%	21.2
	IAC	18.84	88.3%	16.4
	Sn-Plast	19.35	70.9%	14.5
	**Ours**	**13.24**	**100%**	**10.1**
3	Q-learning	15.81	77.4%	11.3
	SARSA	15.98	69.2%	16.3
	IAC	15.69	78.0%	14.6
	Sn-Plast	16.73	74.4%	11.8
	**Ours**	**12.60**	**100%**	**9.8**
4	Q-learning	19.60	82.8%	16.9
	SARSA	20.11	69.5%	15.4
	IAC	18.89	82.2%	13.6
	Sn-Plast	19.62	74.5%	16.8
	**Ours**	**15.69**	**100%**	**11.4**

**Table 3:** Convergence speed of each algorithm in performing dynamic navigation tasks.

Algorithm	Average Number of Explorations Required to Redevelop Navigation Habits			
	Reduce Obstacles	Add Obstacles	Change Starting Points	Change Goals
Q-learning	15.2	13.3	9.8	14.1
SARSA	22.9	13.0	16.4	15.7
IAC	14.9	12.1	10.6	11.8
Sn-Plast	14.3	9.3	11.5	12.2
**Ours**	**1.0**	**1.0**	**1.0**	**10.4**

## Data Availability

The data presented in this study are available on request from the corresponding author.
